# A blockchain based lightweight peer-to-peer energy trading framework for secured high throughput micro-transactions

**DOI:** 10.1038/s41598-022-18603-z

**Published:** 2022-08-25

**Authors:** Nihar Ranjan Pradhan, Akhilendra Pratap Singh, Sahil Verma, Kavita, Marcin Wozniak, Jana Shafi, Muhammad Fazal Ijaz

**Affiliations:** 1 School of Computer Science and Engineering (SCOPE), VIT-AP University, Vijayawada, 522237 Andhra Pradesh India; 2 Department of Computer Science and Engineering, National Institute of Technology Meghalaya, Shillong, 793003 India; 3 Department of Computer Science and Engineering, Chandigarh University, Mohali, 140413 India; 4 Faculty of Applied Mathematics, Silesian University of Technology, 44-100 Gliwice, Poland; 5 Department of Computer Science, College of Arts and Science, Prince Sattam Bin Abdul Aziz University, Wadi Ad-Dawasir, 11991 Saudi Arabia; 6 Department of Intelligent Mechatronics Engineering, Sejong University, Seoul, 05006 Korea

**Keywords:** Mathematics and computing, Computer science, Information technology

## Abstract

With the electric power grid experiencing a rapid shift to the smart grid paradigm over a deregulated energy market, Internet of Things (IoT) based solutions are gaining prominence and innovative Peer To Peer (P2P) energy trading at micro-level are being deployed. Such advancement, however leave traditional security models vulnerable and pave the path for Blockchain, an Distributed Ledger Technology (DLT) with its decentralized, open and transparency characteristics as a viable alternative. However, due to deregulation in energy trading markets, massive volumes of micro transactions are required to be supported, which become a performance bottleneck with existing Blockchain solution such as Hyperledger, Ethereum and so on. In this paper, a lightweight ’Tangle’ based framework, namely IOTA (Third generation DLT) is employed for designing an energy trading market that uses Directed Acyclic Graph (DAG) based solution that not only alleviates the reward overhead for micro-transactions but also provides scalability, quantum-proof, and high throughput of such transactions at low confirmation latency. Furthermore the Masked Authentication Messaging (MAM) protocol is used over the IOTA P2P energy trading framework that allows energy producer and consumer to share the data while maintaining the confidentiality, and facilitates the data accessibility. The Raspberry Pi 3 board along with voltage sensor (INA219) used for the setting up light node and publishing and fetching data from the Tangle. The results of the obtained benchmarking indicate low confirmation latency, high throughput, system with Hyperledger Fabric and Ethereum. Moreover, the effect of transaction rate decreases when the IOTA bundle size increases more than 10. For bundle size 5 and 10 it behaves absolutely better than any other platform. The speedy confirmation time of transactions in IOTA, is most suitable for peer to peer energy trading scenarios. This study serves as a guideline for deploying, end-to-end transaction with IOTA Distributed Ledger Technology (DLT) and improving the performance of Blockchain in the energy sector under various operating conditions.

## Introduction

The energy transaction is making electric power systems increasingly volatile. The supply of renewable energy is changing due to unpredictable sources^1^. At the same time energy consumption is also changing due to sophisticated electric appliances such as electric vehicle and human used devices. Till today we have used traditionally power plant such as fossil based energy to achieve the demand-supply balance. The major challenges in energy sector is scaling back the use of fossil fuels, limit extension of the grids, support new technology for reliable energy supply^1–3^.

A secure electricity trading can be designed using private or public blockchain. Public Blockchain is vulnerable to security threats and various types of attacks. Blockchain has several key features such as decentralization, persistency, anonymity, immutability and security among others. With the current technological development of the internet, energy trading services are moving from offline mode to online mode. Online accessing, storing and maintenance of energy transaction records have various security issues such as scattered and disjoint energy data, interoperability troubles, data security and privacy issues, scalability among other^4–6^. This had paved the path for blockchain based energy trading systems research and implementation. Blockchain inherent characteristics such as decentralized platform, transparency, auditable, and irrevocable digital ledger, etc. attract many organizations to adopt^7,8^. The viable solution to future energy which needs the system to be secure, efficient, decentralization with respect to energy records; digitization with respect to technologies; democratization with respect to more consumer participation and decarbonnization with respect to carbon free green energy resources, is integration of Blockchain Technology^9–11^. Distributed energy system using Blockchain technology can help due to its novel characteristics and can manage the energy transaction efficiently in a real time problem. Despite all these impressive advantages, this technology faces many inherent challenges such as high energy consumption, high transaction confirmation time, low scalability, and privacy issues. To overcome the above mentioned challenges, tremendous research efforts have been underway to a new paradigm such as IOTA the Tangle and Hyperledger framework^12^.

In this paper we investigate the need for adopting IOTA in P2P energy trading in comparison to other Blockchain framework with the following contributions,

- We propose a novel, lightweight, IOTA based community energy trading framework that publishes producer energy data to the Tangle, consumers within same or different community securely subscribes through the Masked Authentication Messaging (MAM) channel and settlement of the transactions is achieved by using IOTA light wallet 2.5.4.
- Performance bench marking of major decentralized ledger technology platforms such as Ethereum, Hyperledger Fabric and IOTA has been considered by taking various parameters such as confirmation time, throughput, effect of payload and bundle size.

In addition, this paper is also enriched with other worthwhile contribution such as:

- The Blockchain enabled energy trading framework implementation and prototype design is presented. *IOTA Tangle* for enacting MAM generated micro-transactions for client communications. A thorough performance evaluation of this prototype is presented herein.

To the best of knowledge of the authors, this paper is the first to present a comprehensive method for Blockchain in energy trading using IOTA. The rest of the paper is organized as follows. “Background” discusses the background related to IOTA DLT, while “Related work” reviews the related work to energy trading. The proposed framework and system architecture has been introduced in “System architecture and proposed framework”. In “Implementation” a detailed implementation procedure has been given. Based on the performance bench-marking the “Performance analysis and discussion” is analyzed. Finally “Conclusion” concludes the paper with a future work.

## Background

The use of IOTA platform with IoT devices i.e., smart meter with PV panels would allow energy producers and consumers to adopt energy transactions in real time with efficient sharing of resources with no fees^13^.

### IOTA framework

**Figure 1:**
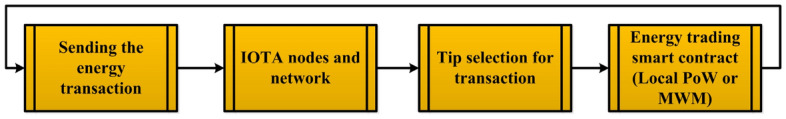
Transaction life cycle in IOTA (P2P energy trading).

IOTA is a distributed ledger technology that is not based on the chain of the blocks as in other Blockchain framework, but through the use of Tangle, a Directed Acyclic Graph (DAG) generated from transactions. It is a distributed ledger with the advantages of scalability, fee less and open source. IOTA provides quantum robust signature for security and Tangle for decentralized network which leads to no single point of failure, no subscription fee for freedom to transact. The transactions represent in DAG are called sites and the issuers of transactions are nodes^14^. Tangle is a public distributed ledger which stores all the transaction in a sequential basis called as snapshot. The addresses used once for sending and signing transaction can not be used again. The Tangle is replicated across all the nodes in IOTA network. Any transaction which is attached into Tangle cannot be changed. The transaction data in a Tangle are made up trytes. The Fig. 1 depicts the transaction life cycle in IOTA^14^.

### IOTA transactions and bundles

A group of transaction signed by a group of node. So, to steal IOTA tokens is very difficult because of bundles transactions. It maintains all transaction from current index to last index.

### Hyperledger framework

Hyperledger Fabric is the most popular permissioned Blockchain tool with its modular architecture. It allows many components such as organizations, peers, channel, orderer, chain code, membership service provider, and certificate authority. An organization refers to a group of nodes called peers where there is no or little trust between separate organizations. Channel is a confidential communication tool between different consortium members. The ordering services are the connection points within and to the network; they handle the transactions invoked from chain code, maintain the global ledgers state consistent, generate blocks, and broadcast them to peers.

### Difference between tangle and blockchain

Although Blockchain and Tangle belong to the same category of distributed ledger technology, they differ with two key points such as; Tangle has no transaction fees and the other is IOTA networks have no miners. Another bottleneck of Blockchain is a collection of sequential chain of blocks, where each block contains a specified number of transactions depending on the block size. So, we can add a new transaction to only to the end of the chain. Whereas in IOTA Tangle which stores the transaction in Directed Acyclic Graphs(DAG) data structure and each transaction is attached to two previous transactions. IOTA is working with the concept to issue a transaction node need to approve two other transactions. In Tangle PoW is not used to secure the network, rather it is used only to discourage spam transactions^14^.

## Related work

There are only a few works that in general focused on Blockchain bench marking in particular on P2P energy trading process. For instance, Hassija et al.^15^ proposed in a distributed IOTA Tangle based energy trading system for unmanned aerial vehicles (UAVs) and charging stations by using tokens. Moreover Shafeeq et al.^16^ conducted an evaluation for IOTA address reuse in an distributed ledger technology by using Cuckoo’s filter approach. The authors in ? has designed an Blockchain enabled multi party healthcare framework using Hyperledger Fabric and Composer. They also designed the access rules for each participants and finally measured the performances using Hyperledger Caliper. From another perspective Park et al.^17^ examined the feasibility of energy trading platform by subjecting to DAG block free data structure. Aside from Fabric and IOTA, Esmat et al.^18^ discussed an Ant-colony optimization method for energy market stakeholders without the need of intermediary and additionally used another layer of Blockchain which provides security through smart contract.

There are several potential uses in the energy field of the Blockchain technology, most of which target P2P energy trade^19^. However, in the field of power sector the parties might alter, electric vehicles, manageable loads carbon storage systems, consumers or prosumers. There are other common Blockchain technology applications to electricity market. The uses of Blockchain in energy may be classified into two major classes: energy trading, certification of clean energy sources. Zhou Su et al.^2^ proposed a contract based permissioned blockchain for secure charging of electric vehicles. They have utilized Byzantine fault tolerance consensus algorithm. Finally, they have compared the efficiency of the proposed system with the conventional schemes. Silvestre et al.^20^ has discussed the blockchain technology in power sector and compared the result taking Tendermint, Ethereum and Hyperledger into consideration. They have also discussed the future applications and perspectives of power blockchain. Murkin et al.^21^ has investigated for peer to peer energy trading market end proposed a novel algorithm that can automate sale and purchase of electricity with optimize market strategy and more consumer centric control. A JavaScript based smart contract simulation was implemented in order to find the performance of the algorithm. Casquicco et al.^22^ has analyzed bibliometrically a review of the decentralized energy trading architectures on Blockchain and IoT. They also reviewed on various proposals on P2P renewable energy trading, power grid model, and combining IoT and Blockchain. Although they have analyzed many Blockchain based peer to peer (P2P) energy trading framework, they have not analyzed any IOTA or Tangle based P2P energy trading. Performance comparison of IOTA, Ethereum, and Hyperledger Fabric in an IoT based application, only qualitatively evaluation has been conducted in literature 14^23^, and^24^. Ferraro et al.^24^ proposed a modification method to IOTA Tangle attachment mechanism that ensures the validation of all transaction in finite time and also preserves the Monte Carlo Selection Algorithm (MCSA). They also presented simulations that validates the results for finite arrival rates. Zhang et al.^25^ proposed a novel control scheme for IoT access by combining IOTA technology with cypher text policy with attribute based encryption. The authors also evaluated the performance of the proposed scheme in terms of throughput for access request and processing. Ren et al.^26^ has presented a solution for task-offloading with a centralized Low-Latency Secure and Reliable Decision Making (LSRDM-EH) algorithm which has an facility of emergency handling. This work is to support the task off-loading for resource constraints edge devices. The security of the network is ensured by providing a blockchain based two layer and multidimensional security. To deal with time-inefficiency problem the authors have proposed a sharding scheme that reduces the system latency. Li et al.^27^ developed a blockchain enabled identity authentication scheme by a new trusted service evaluation model. This model consist of identity authentication scheme and modules related to evaluation submission and publicity. The authenticated users of the proposed system can only submit reviews to vendors. The blockchain network stores the registration, authentication records, user identity and reviews. Xu et al.^28^ proposed a blockchain enabled electric vehicle charging system that avoids third party interference. It also provides a multi-party security between electric vehicles and EVSPs. In this work the digital certificates can be obtained by providing distributed public key infrastructure (PKI) identity and user registration. Wang et al.^3^ has reviewed the role of Unmanned Aerial Vehicle (UAV) in the Space-Air-Ground Integrated Network (SAGIN). Then, three applications of the blockchain-envisioned UAV network are introduced through several classifications. Future challenges and the corresponding open research topics are also described. Based on the aforesaid works, it was concluded that although sizable attempts were made to implement Blockchain based energy trading systems and constituent technologies, yet none of these have accounted for all the following properties, namely smart contract, data privacy and security, prosumer approach as well as presented implementation and performance of their proposed schemes. To this end, the work presented a foolproof, that accounts for all these issues, while presenting the implementation and performance evaluation of the proposed scheme.

**Figure 2:**
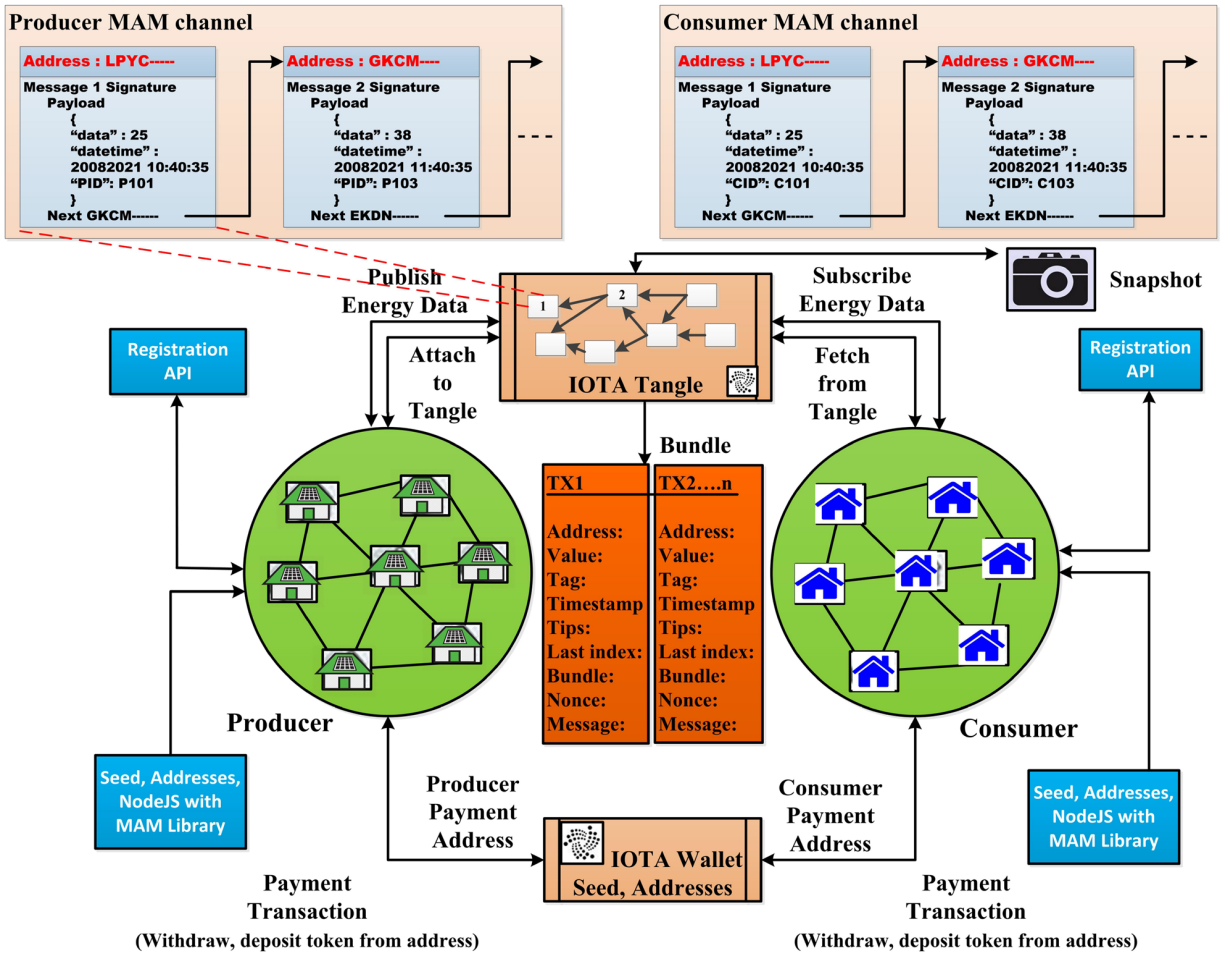
Proposed blockchain based lightweight energy trading process.

## System architecture and proposed framework

In this work we proposed an IOTA blockchain in P2P energy trading system to achieve the effective energy and wallet transaction. Figure 2 is an illustration of the stakeholder’s registration and interaction in an IOTA blockchain network through the API. The Node.js, JOTA library and Masked Authenticated Messaging (MAM) library is used to create IOTA node and network that generates seed, account addresses, MAM channel root and addresses. Then a single or group of producers aggregates their generated energy and fixes the price per unit, per time of the energy and they offer, publish, and attach to the IOTA Tangle through a lightweight MAM channel ID and address. The consumers who are in need of energy subscribes producer MAM channel and fetches the energy data and offer. In return a single consumer or a group of consumers make queries through API about the request for their KWH usage per time. If the offer given by the consumers satisfies their demand, then the flow of energy transaction occurs only after a successful payment is made to the particular IOTA wallet address. This payment reduces their owned token balance. After a producer takes its share against the energy to be transferred, the flow of transaction takes place. The users communicate through an interactive java API over the IOTA network 204. The payment system is governed by an IOTA wallet addresses, namely light wallet 2.5.4. The message published by an producer is considered as a function of payload and next root to point in MAM channel as shown in Eq. (1). The payload is also considered as an integration of transaction data $(TR_{data})$, MAM data $(MAM_{data})$, and auxiliary data $(AU_{data})$. The payload is the integration of energy amount (*KWh*), date, time, participant ID and so forth as shown in Eq. (2). Equations (3), (4), and (5) derives the transaction data $(TR_{data})$, MAM data $(MAM_{data})$, and auxiliary data $(AU_{data})$. $UP_{info}$ is unit price for energy. We conduct the modeling in energy trading environment by assuming producer as a data publisher and published its produced energy amount, date, time and so forth through a MAM channel with his signature on hourly basis. The data along with the next root is attached in a batch wise manner. For publishing the data to Tangle we consider the parameters such as $Network ID, producer ID, random seed, security level_<1, 2, 3>, channel mode_<public, private, protected>,$ and the *callback* i.e. the time interval for publishing data as shown in equation 8. The callback value for the proposed framework is considered as one hour. The proposed work aims to design and deployment of automate the transfer of energy and payment for the usage. The integration of Node.js and java client APIs makes the system easier and more interactive.

1 $$\begin{aligned}&Msg = f\big (payload,\ N_{root} \big ) \end{aligned}$$

2 $$\begin{aligned}&Payload \leftarrow TR_{data} \Vert MAM_{data} \Vert AU_{data} \end{aligned}$$

Energy producer often sells their excessive energy to utilities with a cheaper and fixed price but selling it to the nearby consumers without the intervention of any intermediary. It can impart freedom as well as as the consumption of green energy which is healthy, reliable and secure than that of fossil energy. IOTA facilitates the energy transaction to traceable and immutable. The proposed architecture interconnects the energy source, producer, consumer through a Directed acyclic graph i.e. IOTA network.

3 $$\begin{aligned}&{TR_{data} \leftarrow P_{ID} \Vert C_{ID} \Vert UP_{info} \Vert P_{address} \Vert C_{address} \Vert Sign_{<P,C>}} \end{aligned}$$

4 $$\begin{aligned}&MAM_{data} \leftarrow datetime \Vert channel_{mode} \Vert Channel_{ID} \end{aligned}$$

5 $$\begin{aligned}&AU_{data} \leftarrow Aggr_{kwn} \end{aligned}$$

6 $$\begin{aligned}&EN_{msg} \leftarrow root \Vert Side_{key} \Vert Msg \end{aligned}$$

7 $$\begin{aligned}&DK_{msg} \leftarrow Side_{key} \Vert root \Vert EN_{msg} \end{aligned}$$

The transaction and files moves through a client web browser API then to an Inter Planetary File System (IPFS) and finally attaches the transaction hash values to the Tangle.

8 $$\begin{aligned} Publish Data \leftarrow seed_{<81\ Tryts>} + security\ level_{<1,2,3>} \nonumber \\ +\ Channel_{mode} + callback + NW_{ID}\nonumber \\ // NW_{ID}\ \mathrm {is\ URL\ of\ the\ Testnet/Mainnet} \end{aligned}$$

9 $$\begin{aligned} // PD\ \mathrm {is\ publishing\ energy\ data\ by\ producer} \end{aligned}$$

10 $$\begin{aligned} Subscribe \, Data \leftarrow NW_{ID} + Channel_{mode} + root\nonumber \\ // SD\ \mathrm {is\ subscribing\ data\ by\ consumer} \nonumber \\ Bill \leftarrow TR_{ID} \Vert Usage \end{aligned}$$

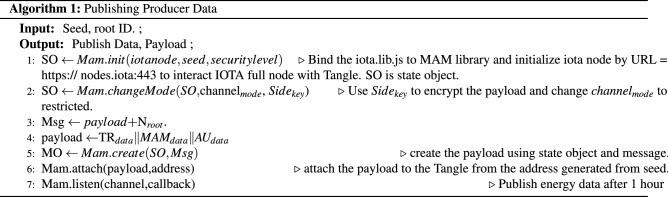

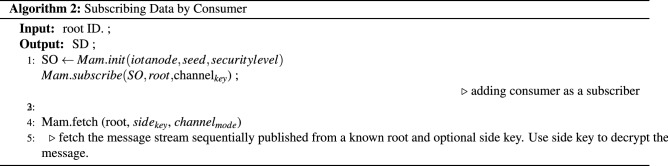

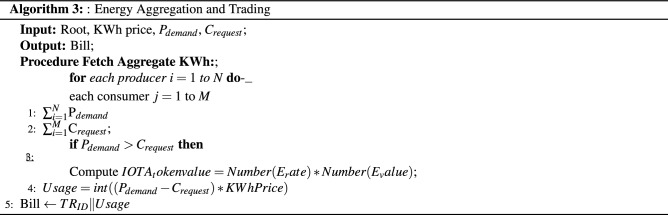


Three algorithms have been proposed for publishing producer data, subscribing data by consumer, and energy aggregation and trading within same or different communities. Finally updating the transactions in consumers and producers externally owned accounts with their seeds and addresses are presented.

- The Algorithm 1 takes the producer IOTA seed and addresses to publish the data to the Tangle. The MAM channel initialization is carried out through the node setup module in iri. The MAM library is bounded with the IOTA java script library. The security level is set as 2, i.e. private and the side key is used for encrypting the producer energy payload, date time, producer ID and so forth.
- Algorithm 2 is used to subscribe and for fetching the data securely from the tangle by providing only channel root address.
- Finally Algorithm 3 is used for the energy aggregation, trading between participant and generating Blockchain records by calculating usages and bills.

## Implementation

In this section the schematic of different modules of implemented Peer to Peer energy trading framework using IOTA blockchain in accordance with the present invention. A user connected to solar panel is an energy producer who sells its energy to consumer devices. A single channel low cost INA219 current/voltage/power sensor that comes in the form of a breakout board and is connected to the Raspberry PI controller pins. A potentionmeter is a variable resistor placed in the circuit to simulate variable power usage. At interval of 1 hour (callback function of MAM) we calculated the average power usage for that period and multiply with the time (one hour) and the energy price in IOTA’s network they both agree upon through smart contract. Finally, after interval of 1 hr, the PI automatically creates a new IOTA transaction and transfers the calculated IOTA light wallet 2.5.4 wallet tokens from the consumer to the producer address before starting a new period. The WiFi module and device controller are used to interface with the IOTA NW.

**Figure 3:**
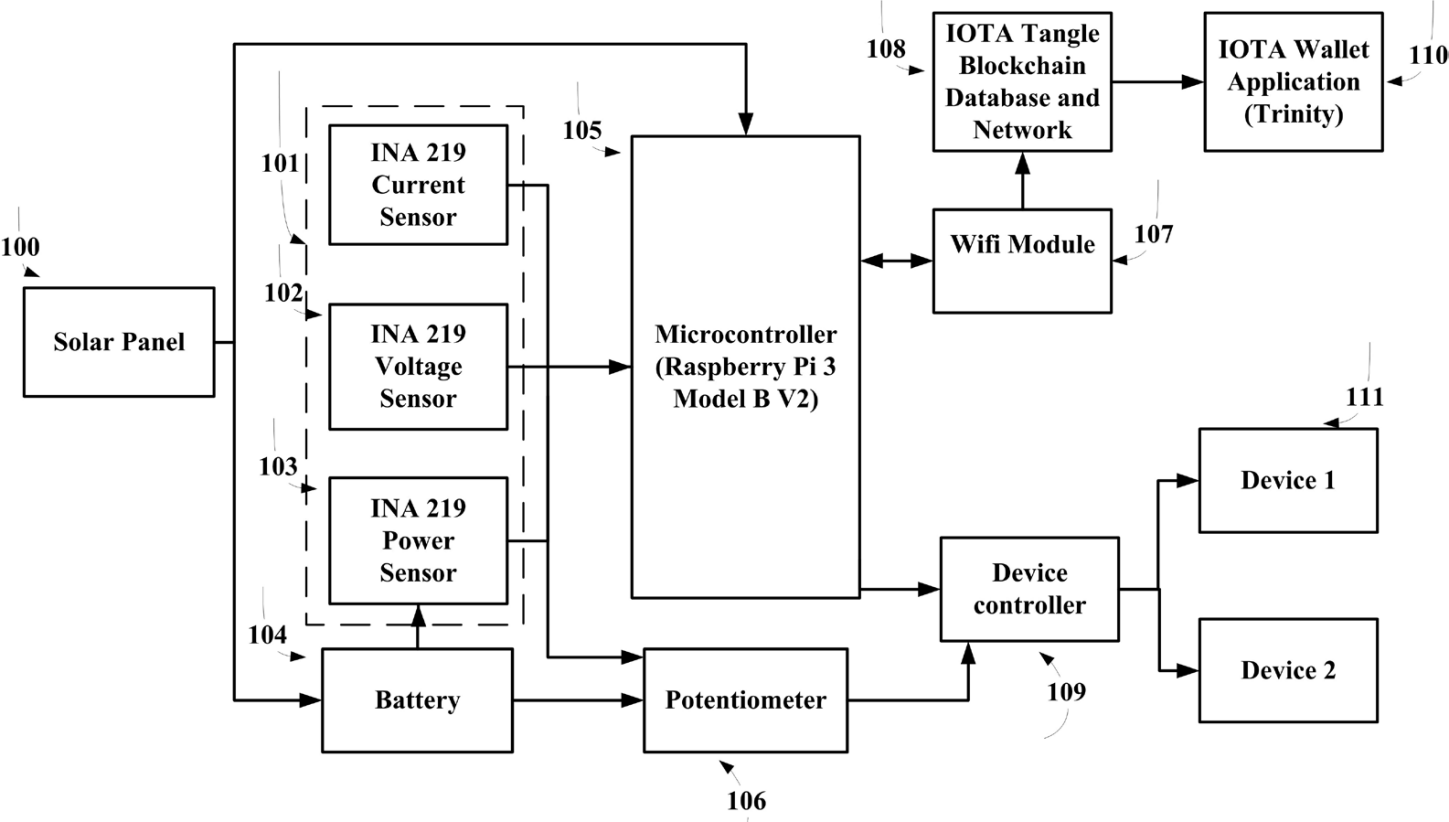
Working of proposed framework elements.

Figure 3 is an illustration of the schematic of different modules of claimed Peer to Peer energy trading framework using IOTA blockchain in accordance with the present invention. A user connected to solar panel 100 is an energy producer who sells its energy to consumer devices 111. For our invention we used a small panel capable of charging our 12 V rechargeable battery. 101, 102, and 103 is single channel low cost INA219 current/voltage/power sensor that comes in the form of a breakout board and is connected to the Raspberry PI controller pins 105. A potentiometer 106 is a variable resistor placed in the circuit to simulate variable power usage from battery 104. We used a 12 V small battery 104 with enough voltage to light up a bulb or charging mobile at the consumer side. Then, at interval of 1 h we calculated the average power usage for that period and multiply with the time (1 h) and the energy price in IOTA’s network 108 they both agree upon through smart contract. Finally, after interval of 1 h, the PI automatically creates a new IOTA transaction and transfers the calculated IOTA Trinity wallet tokens 110 from the consumer to the producer address before starting a new period. The Wi-Fi module 107 and device controller 109 are used to interface with the IOTA NW. First we take a single Raspberry PI 3 with an attached power monitoring sensor and puts it in the power circuit between the producer batteries and the consumer lights. Then, every second we have the PI take a reading from the sensor and log the current power usage. Then, at some predefined period of time (i.e. 1 h for our proposed work) we calculate the average power usage for that period and multiply with the time (one hour) and the energy price in IOTA’s they both agree upon. So all simulation does not pass through the PI 3. Finally, after each period (1 h), the PI automatically creates a new IOTA transaction and transfers the calculated IOTA tokens from the consumer to the producer address before starting a new period. The Tables 5, 6 and 7 in the result section has been revised that represents the Raspberry PI 3 estimated time (in s) to create payload, attach to the tangle, and fetch data from the tangle. The JavaScript code for this work contains some important variables such as pay frequency variable defines the period in seconds from where we calculate the average power consumption and issues the IOTA payment transaction. The mW price variable specifies the price of IOTA’s per milliwatt/second (mW/s) of energy as shown in listing 4. Any decimals from the calculated IOTA transaction value are removed as we cannot send fractions of an IOTA.

**Table 1:** Specifications and hardware requirements for comparison of proposed P2PET

CPU	Quad Core (3 nos.)		
RAM	8GB		
4*OS	Ethereum	Ubuntu 18.04 (64 bit)	
	2*IOTA	Sender	Rasbian
		Receiver	Ubuntu 18.04
	Hyperledger Fabric	Ubuntu 18.04, VS code 1.39	
4*Software	Ethereum	GETH 1.9.2 and Caliper	
	2*IOTA	Sender	IOTA MAM Client / JOTA library
		Receiver	IOTA Hornet, IRI 1.6.0 and light wallet 2.5.4
	HF	Blockchain Platform extension 1.0.3 and Caliper	
Database	RockDB (for IOTA) and GolevelDB or CouchlevelDB (for Hyperledger Fabric)		
Sensors	Voltage/current/power	INA 219

**Table 2:** Case I: energy trading simulation between two different community.

Producer ID	Produce energy in (KW)	Tip selection priroty	Unsold energy	IOTA token balance before	IOTA token balance after	Consumer ID	Request energy in (KW)	IOTA token balance before	IOTA token balance after
P101	5	4	5	0	0	C101	5	500	450
P102	20	1	0	0	200	C102	7.5	500	425
P103	19	2	0	0	190	C103	7.5	500	425
P104	2.5	5	2.5	0	0	C104	7.5	500	425
P105	12.5	3	1.5	0	110	C105	7.5	500	425
P106	1.5	6	1.5	0	0	C106	10	500	400
P107	1	7	1	500	500	C107	5	500	450

**Table 3:** Case II: energy trading simulation within the same community.

Producer ID	Remaining energy in (KW)	Tip selection priroty	Unsold energy	IOTA token balance before	IOTA token balance after	Consumer ID	Request energy in (KW)	IOTA token balance before	IOTA token balance after
P101	5	1	0	0	50	P107	10	500	400
P104	2.5	2	0	0	25	0	0	0	0
P105	1.5	3	0	0	15	0	0	0	0
P106	1.5	4	0.5	0	10	0	0	0	0

### Setting up an IOTA node and seed generation

**Figure 4:**
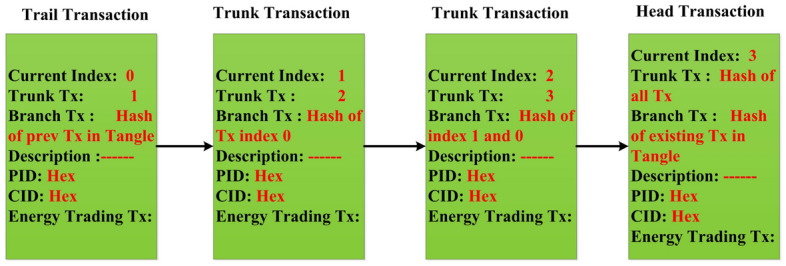
IOTA bundle structure.

**Figure 5:**
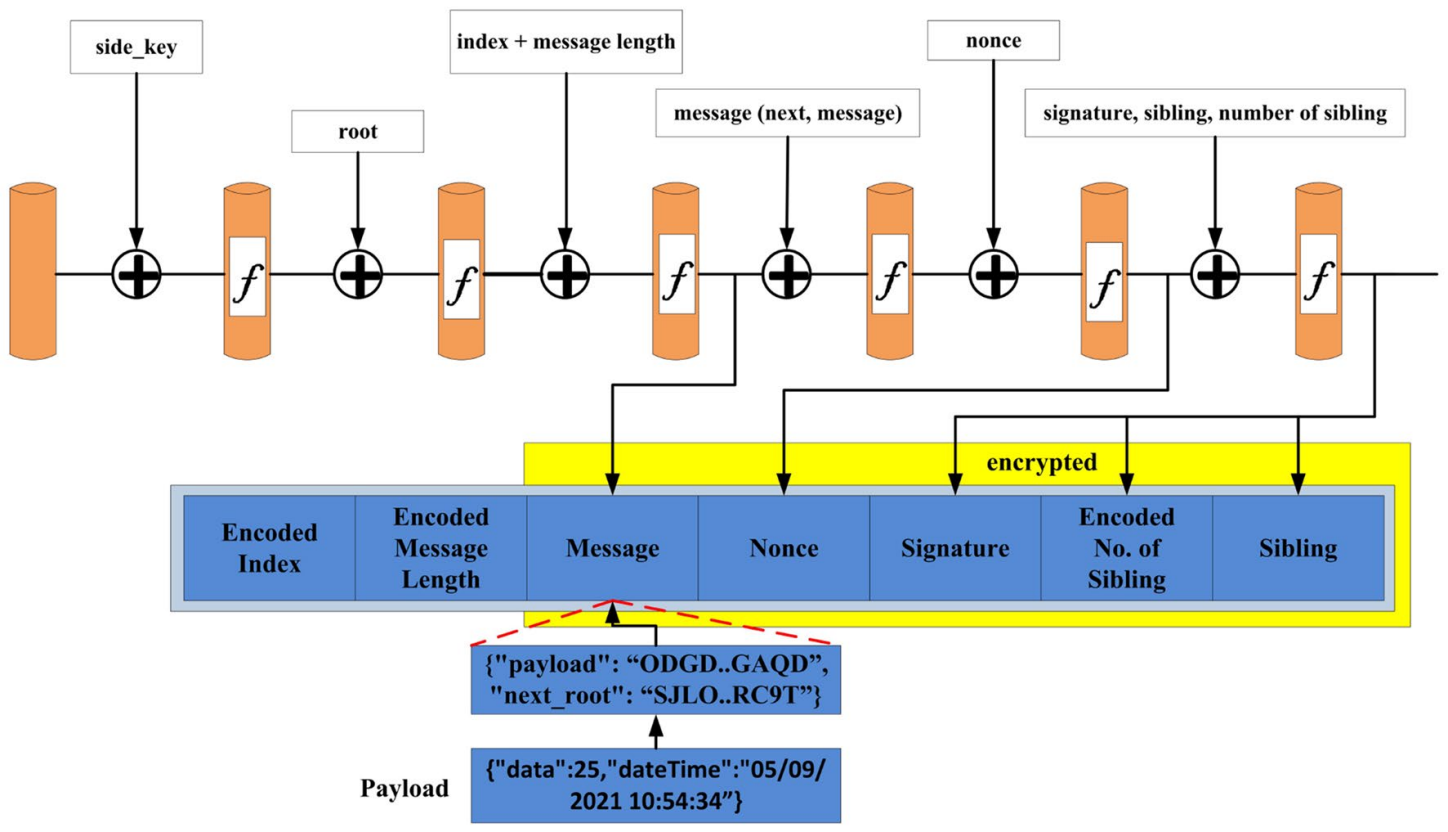
Masked payload energy data of consumer.

The IOTA node has been installed on a local machine. The IOTA node has integrated with the energy trading application. Although IOTA supports many languages, we considered Java. The *Openjdk* version 10.0.2 has installed in an *Ubuntu 18.04.4* machine for the proposed application. Then we run the IOTA node after installing the *iri java* file. IOTA node is configured by setting the parameters in the *iri.ini* and a script for starting the IOTA network. The complete requirements with their specification are shown in Table 1 Finally when the node is running, we found the db and logs of that node has created. It provides two basic implementation specification one is iri and another is IOTA wallet. To execute the java archive we created a script file named as start.sh. we used the screen command to create node in console mode in the background. The basic configuration in *iri.ini* is set by setting the IRI port number to 14265, *UDP_RECEIVER_PORT=14700, neighbors, ixi_dir, headless, debug, testnet and db_path*. IRI port is configured so that the node is going to expose all its interfaces. The UDP receiver port is used to receive the connection from its peers to sync up its Tangle node. The neighbor’s configuration has the address of neighbor of peers that node needs to create a synced up database called tangle. The *IXI* directory is the directory of extensions like messaging that IOTA provides. The *HEADLESS* flag indicates that we are not running the nodes to be connecting to a ledger wallet. Listing 1, 2, 3 shows how the neighbours are added, attached to Tangle, and broadcast-ed to all node. Figure 4 shows a sample of IOTA transaction bundle structure. Similarly Fig. 5 refers to designing Masked Payload Energy Data of Consumer. To interact with IOTA network these commands are executed. We found the log files by tailing them.

### Deployment of nodes and address generation

The *Node.js, Rock DB* and IOTA nodes have been installed. To run the client application the *Javascript* and *React.js* are installed. Deployment of the application is done in our local network. Both *Hornet* and *Java IRI* software are installed to run the nodes in IOTA network. It provides the facility to read and write access to the Tangle which also validates the transaction by the nodes and finally stores the transaction to the ledger. Other prerequisites are a dual core CPU, 4GB RAM, public IP address, storage docker and some other ports for internet. To, get the latest snapshot we migrated from IRI to hornet. To receive the transaction we create address. The function used for address generation are $gen_new_address(index, \,count, \, security \, level)$. Index is the starting value for address generation. Count is the number of addresses that will be generated. We consider the security level as 2 for resulting multi-signature in the transaction.
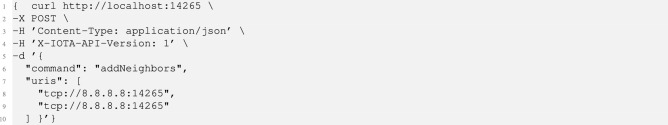

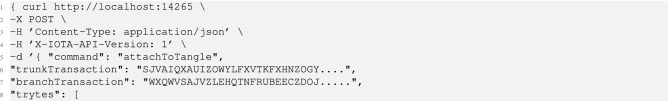

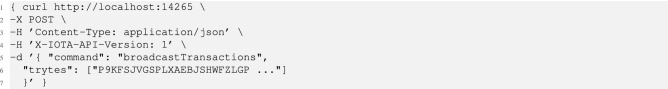

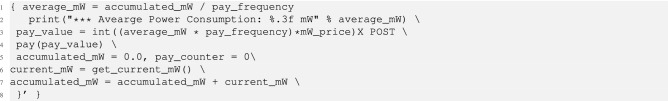

**Figure 6:**
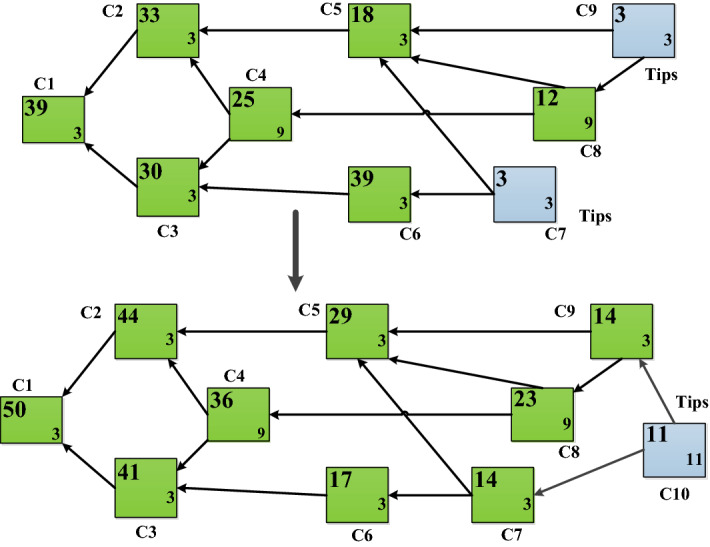
DAG before and after assigning the new transaction issued by node C10.

Table 2 indicates scenario of energy trading between two communities has been simulated. The participant in the producer group has to broadcast or request their amount of energy to sell. Although they produces a sum of 66.5KW, but they have to sell only 50kw to consumers on the specified date and time. The IOTA Tip selection strategy is will consider the highest value (KW) first. As you can see, we have only considered 20KW of P102, 19KW of P103 and 11KW of P105 for selling. Figure 6 shows Tangle structure of our proposed model, where node C9 and C7 are the Tips before the arrival of new transaction C10. The shown DAG for the proposed framework can generate cumulative weight of 50. Consider each of the participant in producer group have 0 tokens as a balance except P107. After energy trading is completed the energy transaction and payment of IOTA tokens are updated in the Table 2. Similarly, Table 3 indicates energy trading within the same community. Although P107 is producer and produces very less amount of energy and need 10KW more amount of energy. It is observed from the Table 2 that the rest amount i.e., 11.5 KW is available to trade among themselves in producer community. Participants 107 of the producer wants to buy 20KW with the same condition. After 50KW trading is over the participants P101, P104, P105, P106 have balance of 5 KW, 2.5 KW, 1.5 KW, 1.5 KW. So, the energy transaction of 10 KW against 100 token takes place. Finally, the values are updated in the Table 3.

**Figure 7:**
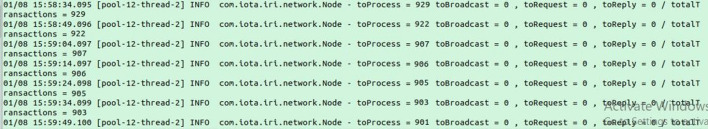
IoTA network node processing.

**Figure 8:**
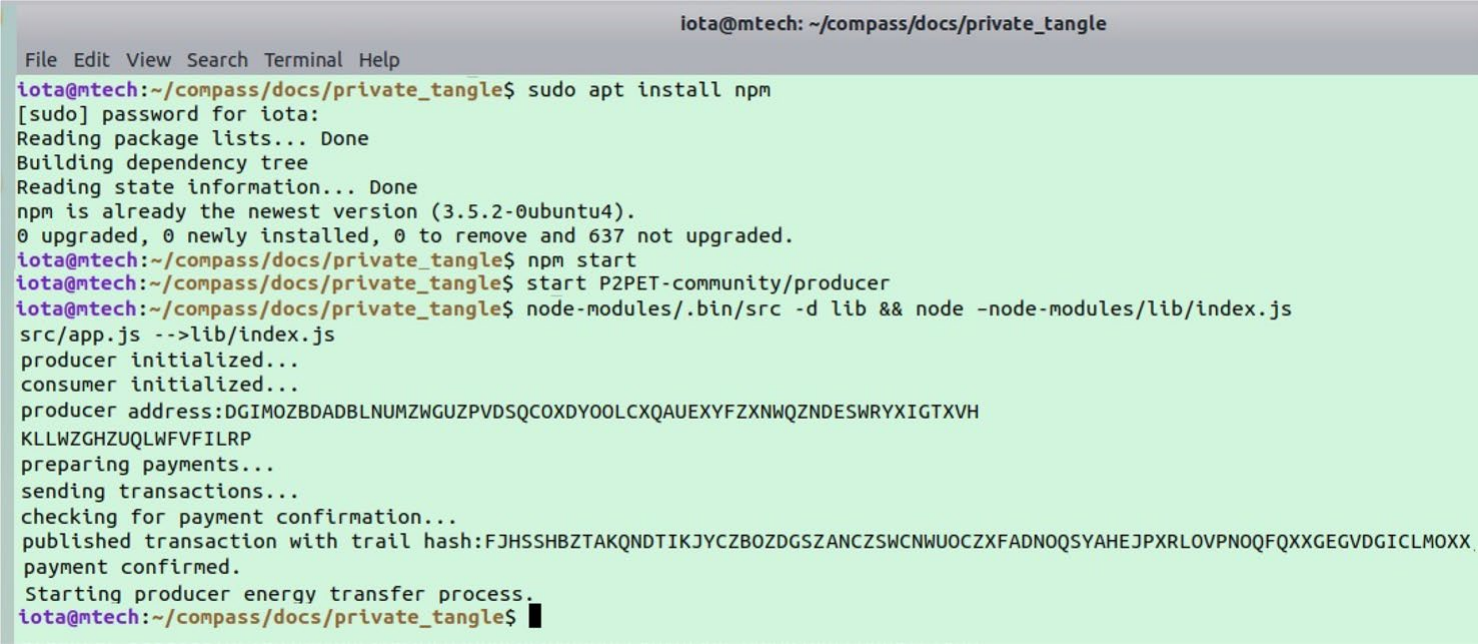
Producer, consumer token transfer.

**Figure 9:**
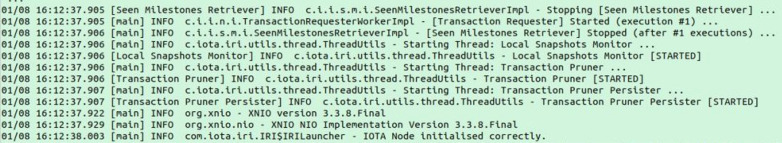
IoTA node initialization and milestone retrieve.

**Figure 10:**
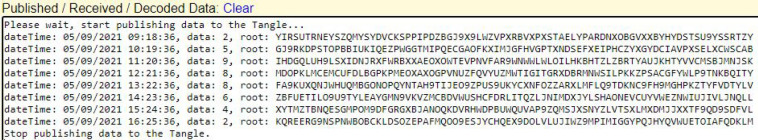
Publishing producer data to tangle.

**Figure 11:**
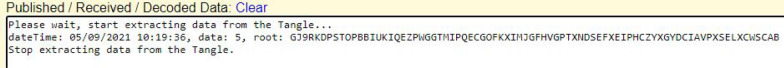
Consumer retrieving from tangle.

**Table 4:** Summary of performances evaluated for proposed P2PET IoTA nodes.

Hostname	SSL	IRI-version	SyncStatus	Free memory)	Neighbors	% node health
https://nodes.iota.producer:443	Yes	0.5.8	3872/3872	0MB	32	100 %
https://nodes.iota.consumer:14267	Yes	0.5.8	3872/3872	0MB	37	100 %

**Table 5:** Create payload.

Payload size (in KB)	100	300	500	700	900
Create time	0.4 to 0.55	0.4 to 0.65	0.5 to 0.74	0.8 to 0.88	1 to 1.45

**Table 6:** Attach time.

Payload size (in KB)	100	300	500	700	900
Attach time	1 to 32 s	4 to 38 s	3 to 40 s	2 to 35 s	2 to 28 s

**Table 7:** Fetch time.

Payload size (in KB)	100	300	500	700	900
Fetch time	0 to 5 s	0 to 8 s	10 to 35 s	15 to 45 s	20 to 48 s

### Setting up energy trading application by Java client (API)

We run IOTA nodes on Raspberry Pi 3 as a light node along with current and voltage sensor (INA219) for publishing to and fetching data from the Tangle. IOTA provides IOTA java which is a client API that is a thin wrapper over the JSON RPC calls. To interact with the energy trading IOTA application the API was run. Eclipse is used to setup the java IOTA client (JOTA) and ran the API through some test cases. Iota java is installed which is a repository of IOTA ledger. To interact with IOTA, we build an address through Trinty wallet which generates seeds. Further, we supplied the protocol i.e., https, host name and the port, as it is running locally. Provided the local PoW through Perl driver which is an engine that generates POW locally. Created the client separately where introducing the SSL certificate. To generate an address an IOTA needs a seed. Seed is like a password or a private key that maps to an address. We received the hashes for the tips. It is found out the effect of transaction by submitting to the API. From the Tangle explorer, we collected an address for which there is a transaction. To retrieve the transaction details like hash, address, timestamp, index values, transaction bundle, branch and trunk transaction, nonce, has been added with a list of addresses. getInputs function returns the combined balance of all the addresses available for our seed. It also transfers IOTA from one seed to the other. Passed the tips to find the milestone transaction hash. Attach to Tangle is an API that is needed to execute PoW and submitted.

### Setting and integrating IOTA network

IOTA private network is designed, which is a collection of interconnected nodes, and each node stores a copy of the Tangle through a Hornet plugins. The energy trading private network essential means any access to the Tangle requires permission. Testing of our energy trading application conducted in a local environment. Retrieval of data at any time in future and confirming the signed transaction has been done. The timestamp is attached at which this transaction was sent.

## Performance analysis and discussion

In this section we analyzed the performances of our proposed system. The scenarios of our proposed P2P energy trading system has been experimented using hardware devices such as solar panel, potentiometer, voltage/current sensor and IOTA infrastructure. The reason behind taking trade window as 1hr is to get maximum power from the solar panel. The trade window can be made as small as 5 minutes, if the supported hardware is with multiple number of solar panels. Getting real time hardware sets at present is beyond the scope of the authors. Figure 7 depicts the node processing in a IOTA Framework. Figure 8 depicts the final output of the smart contract where producer and consumers are initialized, and creates an address for energy and token data transactions. Figure 9 represents P2PET framework initialization. Similarly separate number of permission-ed nodes are created for producer and consumer by specifying the URL in the light wallet 2.5.4. The Host name, synchronous status, number of neighbour nodes etc. are shown in Table 4. The energy data collected from the Poly-crystalline Solar Panel, and voltage sensor (INA321) have been published to and subscribed from Tangle as shown in Figs. 10 and 11. The Table 5 represents the effect of bundle create time with respect to increasing payload size. Similarly Table 6 represents the effect of attach time to tangle (for producer publishing their data to MAM) with the increasing order of their payload size. Table 7 represents the and effect of fetch time to tangle (for consumer subscribing the same data from MAM) with the increasing order of their payload size. The other parameters are also analyzed as mentioned below.

### Effect of payload and bundle size

We use MAM to store messages payload of different sizes on the Tangle to investigate the time requirement. The messages are stored on the tangle by creating transaction bundle containing multiple transaction bundle. Each signature message fragment field of these transaction objects contains part of the file.

11 $$\begin{aligned} \textit{Message \, (1\, MB)}= & {} \textit{1,764,208 \, Trytes} \end{aligned}$$

12 $$\begin{aligned} \textit{Transaction \, Bundle\, (TB)}= & {} \frac{(1764208)}{(2187)} Trytes = \textit{807\, Transaction\, Objects} \end{aligned}$$

**Figure 12:**
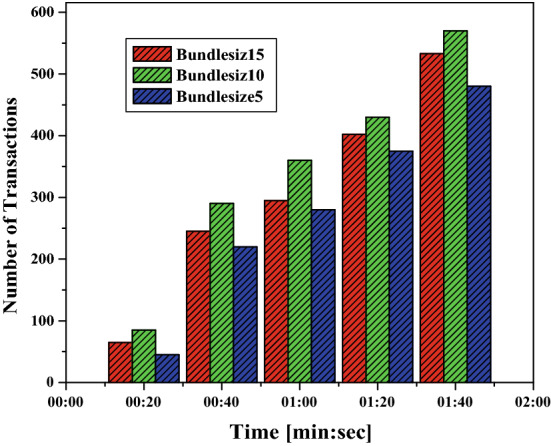
Effect of payload and bundle size with transaction.

Figure 12, depicts that more spikes are marked with bundle size 10 i.e. a greater number of transactions per second compared to a smaller number of transactions with bundle size 5 and 15.

**Figure 13:**
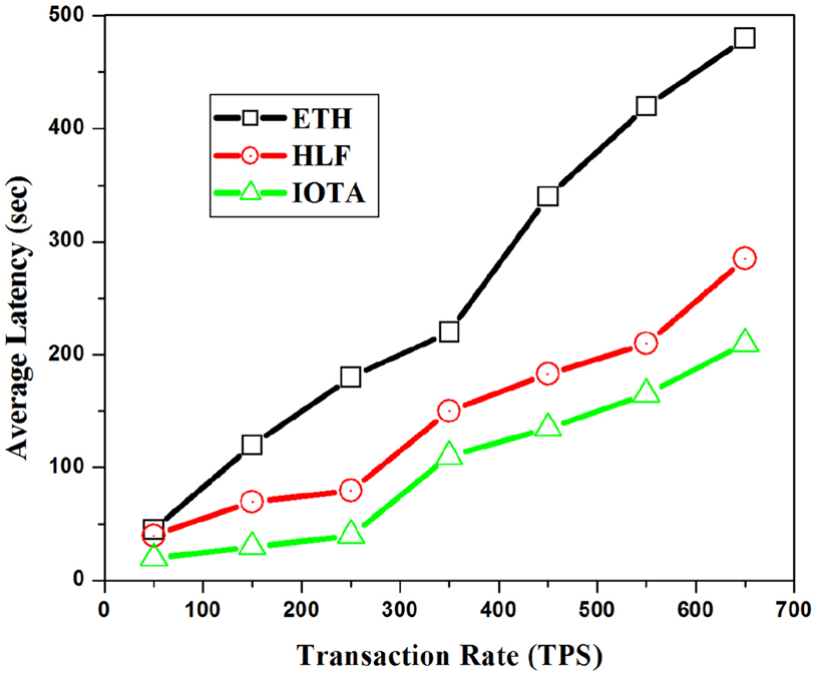
Confirmation latency with varying transaction rate.

**Figure 14:**
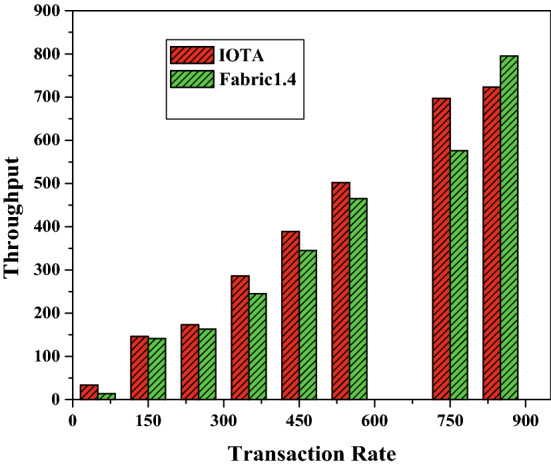
Throughput.

**Figure 15:**
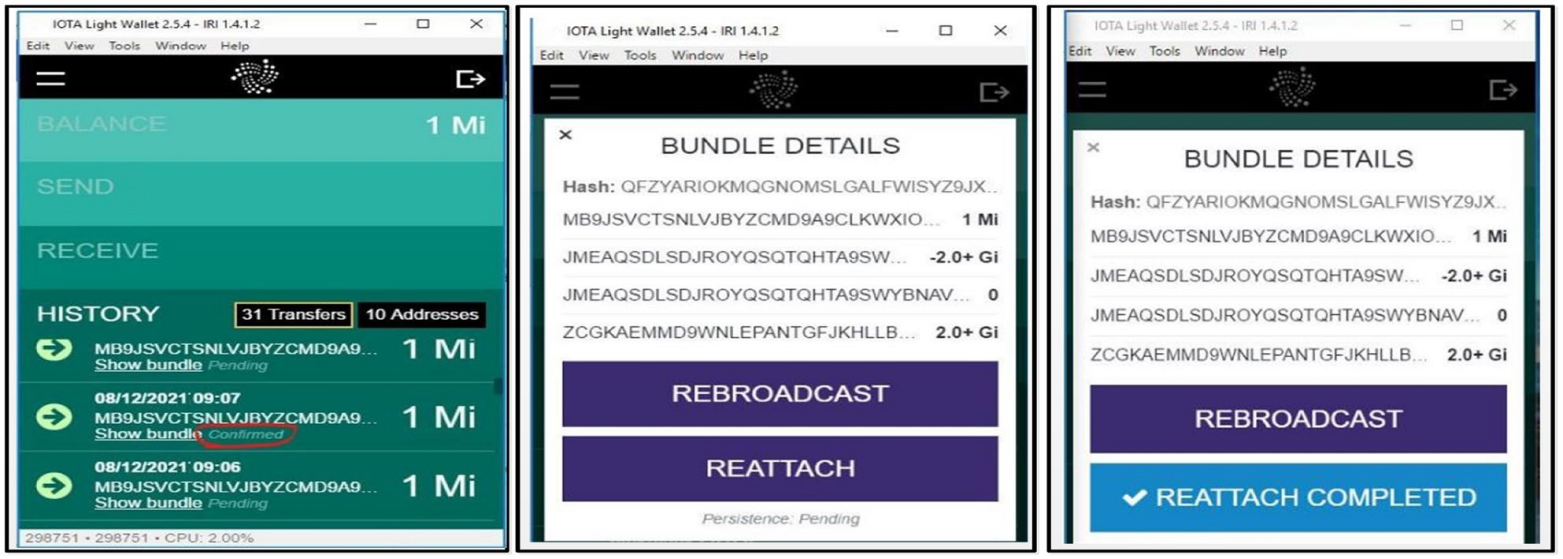
Confirmed and pending transactions, bundle details and reattaching the transactions in IOTA light wallet 2.5.4 IRI.

### Confirmation latency

The confirmation latency is defined as the the number of transaction confirmed and appended to the ledger by the network and the number of transaction submitted for processing. The confirmation latency can be calculated as in Eq. (11):

13 $$\begin{aligned} \textit{Confirmation\, latency}_{(B)}= & {} \frac{\sum _{i=1}^{n} (T^{i}_{confirm} - T^{i}_{submit})}{Num(Trans,\Delta t)} \end{aligned}$$

where n $=$ Num(Trans,t). Most energy trading scenarios put forward strict demands on confirmation latency. Figure 13 indicates the confirmation time of IOTA is substantially reduced compared to that of fabric and Ethereum network. It indicates the latency for querying and writing success rate of transactions in Fabric 1.4 is lower than in IOTA. Also, as is usual, latency increased as the system scaled up with more nodes and network size.

### Throughput

Throughput is defined as the number of transaction per second in a blockchain network.The unit of throughput is transactions per second (TPS). The throughput can be calculated as in Eq. (13):

14 $$\begin{aligned} \textit{Throughput}_{(B)}= & {} \frac{Num(Trans, \Delta t)}{t_{i}-t{j}}TPS \end{aligned}$$

Figure 14 shows the throughput against the transaction rate. Throughput of IOTA network is measured as highest whereas it is reduced for Fabric 1.4 when the number of transaction grows from time to time. Similarly Fig. 15, represents, how the pending transactions in the bundles are confirmed, the bundle structure and reattaching the transaction into the tangle.

## Conclusion

In this paper, a Blockchain-based lightweight community energy trading framework using IOTA as the distributed ledger has been proposed. A Tangle data structure and bundles are used to deal with trail, trunk and head transactions of MAM channel. Comparison and discussion of the performance benchmarking with respect to Hyperledger Fabric, Ethereum and IOTA-assisted energy trading scenario has been done. By analyzing the experimental results, we focus on energy data publishing and subscribing by producer and consumer respectively by a secure lightweight channel. Moreover, we modify the implementation of IOTA in terms of attaching data to a IOTA Tangle and fetching from it through MAM channel and authentic signature. The future work of the proposed energy trading model is to separately implementing smart contracts for all these market can have a high impact on the performance, security and scalability of the overall system. Moreover the energy transactions has to go through a consensus process and validated by validators before adding it to the blockchain network leading to a slow response time and costly operation.
