# Author Correction: A blockchain based lightweight peer-to-peer energy trading framework for secured high throughput micro-transactions

**DOI:** 10.1038/s41598-022-25504-8

**Published:** 2022-12-05

**Authors:** Nihar Ranjan Pradhan, Akhilendra Pratap Singh, Sahil Verma, Kavita, Marcin Wozniak, Jana Shafi, Muhammad Fazal Ijaz

**Affiliations:** 1 School of Computer Science and Engineering (SCOPE), VIT-AP University, Vijayawada, Andhra Pradesh 522237 India; 2 Department of Computer Science and Engineering, National Institute of Technology Meghalaya, Shillong, 793003 India; 3 Department of Computer Science and Engineering, Chandigarh University, Mohali, 140413 India; 4 Faculty of Applied Mathematics, Silesian University of Technology, 44-100 Gliwice, Poland; 5 Department of Computer Science, College of Arts and Science, Prince Sattam Bin Abdul Aziz University, Wadi Ad-Dawasir, 11991 Saudi Arabia; 6 Department of Intelligent Mechatronics Engineering, Sejong University, Seoul, 05006 Korea

Correction to: *Scientific Reports* https://doi.org/10.1038/s41598-022-18603-z, published online 25 August 2022

The original version of this Article contained an error in Affiliation 1, which was incorrectly given as ‘School of Computer Science and Engineering (SCOPE), Vellore Institute of Technology, Vijayawada 522237, Andhra Pradesh, India.’

The correct affiliation is listed below:

School of Computer Science and Engineering (SCOPE), VIT-AP University, Vijayawada 522237, Andhra Pradesh, India.

